# Stochastic resonance in vestibular perceptual thresholds for translation but not rotation

**DOI:** 10.1007/s00221-026-07417-4

**Published:** 2026-11-10

**Authors:** Raquel C. Galvan-Garza, Ajitkumar P. Mulavara, Charles M. Oman, Torin K. Clark

**Affiliations:** 1 Human Systems Laboratory, Dept. of Aeronautics and Astronautics, Massachusetts Institute of Technology, Cambridge, MA USA; 2 Jenks Vestibular Physiology Laboratory, Massachusetts Eye and Ear Infirmary, Boston, MA USA; 3 KBR/HHPC2, Houston, TX USA; 4 Bioastronautics Laboratory, Smead Aerospace Engineering Sciences Dept, University of Colorado, Boulder, CO USA

**Keywords:** Noisy galvanic vestibular stimulation (nGVS), Tolith organs, Semicircular canals, Motion perception

## Abstract

Stochastic resonance, in which adding a certain amplitude of noise improves sensory information transfer, has been observed in vestibular perceptual thresholds, particularly for roll tilts. The semicircular canals and otolith organs are both stimulated during roll tilt, so it is yet to be determined whether vestibular perceptual stochastic resonance involves the canals or otoliths (or both). Animal studies have shown galvanic vestibular stimulation (GVS) stimulates both the canal and otolith afferent neurons. In one human study, noisy GVS (nGVS) at a single subject-specific amplitude improved thresholds for translations (stimulating the otolith organs), but not for rotations (primarily stimulating the canals). However, these studies did not investigate the exhibition of stochastic resonance with nGVS, which requires identifying a dip in thresholds with increasing amplitude of nGVS. We tested human thresholds for roll rotation about an Earth-vertical axis while the subject was supine (canals) and inter-aural translation in an Earth-horizontal axis while seated upright (otolith organs), each while applying a broad range of different nGVS amplitudes (0 to ± 700 µA peak-to-peak) to identify the presence of stochastic resonance (i.e., the theoretical shape of threshold improvement with an optimal amplitude of nGVS). Six of 11 subjects (55%) exhibited stochastic resonance for inter-aural translation by our most rigorous criterion (significantly higher proportion than expected with no underlying stochastic resonance based upon simulations, *p* = 0.001), in contrast to only 1 of 12 subjects (8.3%) for roll rotation (*p* = 0.239). We conclude stochastic resonance in vestibular perceptual thresholds using nGVS is much more common for otolith-mediated motions (e.g., translation), as compared to canal-mediated rotations. Thus, nGVS can extend the operating range of the otolith-mediate perceptual system in some individuals.

## Introduction

Stochastic resonance (SR) is a phenomenon in which the response of a non-linear system to an input signal is benefited by the presence of a particular non-zero amplitude of noise (for reviews see Gammaitoni et al. 1989; Collins et al. 2003; Moss 2004, McDonnell and Abbott 2009; Aihara et al. 2010). SR has been described broadly as “noise benefit” (McDonell and Abbott 2009). The signature of SR is a pseudo-bell shape performance curve as a function of the noise amplitude being added, with a peak in performance at some optimal noise amplitude (Moss 2004; Voros et al. 2022). Enhanced perception has been demonstrated via SR in several sensory channels including visual (Piana et al. 2000; Riani and Simonotto 1994; Simonotto et al. 1997, 1999), auditory (Ries 2007; Zeng et al. 2000), and tactile (Collins et al. 1996, 1997; Enders et al. 2013; Richardson et al. 1998).

Further, studies have found vestibular responses are improved with the addition of noisy galvanic vestibular stimulation (nGVS, also referred to as stochastic vestibular stimulation or SVS). For example: postural sway can be reduced with nGVS (Mulavara et al. 2011a, b; Goel et al. 2015; Inukai et al. 2018a, Fujimoto et al. 2018); the vestibulospinal reflex is enhanced (Wuehr et al. 2018): locomotor function improved (Mulavara et al. 2015), and learning of locomotion enhanced (Putman, et al. 2021). Previous studies have also demonstrated SR improvements in vestibular reflexes in patient populations (Iwasaki et al. 2014, Wuehr et al. 2016; Schniepp et al. 2018, Fujimoto et al. 2018) or older adults (Fujimoto et al. 2016, Inukai et al. 2018b), although other studies did not find significant effects (Assländer et al. 2021; Fujimoto et al. 2025). Notably, our study (Galvan Garza, 2016and the present paper) addressed SR improvements in vestibular perceptual thresholds due to nGVS in normal, healthy, young participants. The present study compares the exhibition of SR using nGVS in translation and rotational vestibular perceptual thresholds.

Two studies identified improvements in roll tilt (stimulating both the otoliths and the canals) vestibular perceptual thresholds amongst healthy individuals (Galvan-Garza et al. 2018; Keywan et al. 2018). Keywan et al. (2018) found a subject-specific amplitude of nGVS improved roll tilt thresholds as compared to sham, using roll tilt motions with single-cycle sinusoids in angular acceleration of 0.5–1 Hz (2 and 1 s long motions, respectively), but found no effect for 0.2 Hz roll tilts (5 s long motion). Interestingly, using 0.2 Hz roll tilts, we (Galvan-Garza et al. 2018) found enhanced thresholds with nGVS. Specifically, Galvan-Garza found significant improvements on average across all subjects when applying 300 uA, as well as 500 uA (defined roughly as peak amplitude, see Table 1 in Galvan-Garza et al. 2018), as opposed to only with a subject-specific optimal nGVS amplitude. Critically, the observed pattern of improvement was consistent with the characteristic SR bell-shaped curve, as determined by blinded judges, suggesting the mechanism of enhanced roll tilt thresholds with nGVS was via SR.

**Table 1:** Upright roll tilt, supine roll rotation, and inter-aural translation comparison. Data are presented on a subject by subject basis to enable any future analysis

Subject Codes		Motion	Optimal nGVS amplitude (𝝁A)	% Change in Threshold
Previous study	This study			
S1	S1	Upright Roll Tilt	300	-46.0
		Supine Roll Rotation	-	-
S3	S6	Upright Roll Tilt	500	-10.4
		Supine Roll Rotation	300	-24.8
		Inter-aural Translation	500	-28.5
S4	S7	Upright Roll Tilt	500	-34.3
		Supine Roll Rotation	-	-
		Inter-aural Translation	300	-18.7
S5	S8	Upright Roll Tilt	300	-14.8
		Supine Roll Rotation	-	-
		Inter-aural Translation	500	-63.1
S6	S9	Upright Roll Tilt	300	-5.4
		Supine Roll Rotation	-	-
		Inter-aural Translation	500	-45.0
S10	S10	Upright Roll Tilt	200	-26.4
		Supine Roll Rotation	-	-
		Inter-aural Translation	500	-33.4
S12	S12	Upright Roll Tilt	300	-40.6
		Supine Roll Rotation	200	-24.9
		Inter-aural Translation	-	-

Roll tilt motions, about an Earth-horizontal axis, stimulate both the otoliths (due to the change in the direction of gravity) and the semicircular canals (due to the angular rotational velocity) of the vestibular system (Lim et al. 2017). Thus, there remains uncertainty whether the benefits of nGVS in vestibular thresholds are due to perceptual processes which are canal-mediated, otolith-mediated, or both. In the dark, passive roll tilt motions also provide non-vestibular cues, however, these thresholds are dramatically elevated in patients with bilateral vestibular ablation (Valko et al. 2012a, b), so are often referred to as ‘vestibular’ perceptual thresholds.

GVS (including nGVS) applied by surface electrodes is non-specific in its target and both semicircular canal and otolith afferent neurons are stimulated (Goldberg et al. 1984, Kwan et al. 2019; Forbes et al. 2023). However, it remains unclear whether nGVS induces SR effects via canal, otolith, or both physiological sensory systems. Most studies of nGVS focus on postural sway and balance (Mulavara et al. 2011a, b; Goel et al. 2015), which involve both the canals and otoliths. A study of ocular counterroll during whole body upright roll tilts found that nGVS increased the ocular response at frequencies from 0.03 to 0.25 Hz (Serrador et al. 2018). Although discussed as an otolith specific response, the ocular counterroll in Serrador et al. could be partially influenced by the canal stimulation, specifically at the higher upright roll tilt frequencies tested.

Thus, it is important to understand by which vestibular sensory pathway (or both) nGVS enhances vestibular perceptual thresholds, since roll tilt motions produce canal and otolith cues. To our knowledge, only one published study has aimed to investigate the canal vs. otolith role in nGVS improving vestibular perceptual thresholds. Specifically, Keywan et al. (2019) tested yaw rotation thresholds (with the head tilted forward at the neck by ~ 71 degrees) to primarily stimulate the semicircular canals, as well as Y-axis (interaural) horizontal translation thresholds stimulating the otoliths. Subjects received a subject-specific optimal nGVS amplitude (or sham, in randomized order) for threshold testing, defined as that which best reduced each subject’s postural sway in a balance test on foam. Keywan et al. (2019) found Y-translation thresholds improved by 38.8% with nGVS as compared to sham (*p* < 0.03), but found no significant change for yaw rotation (with 71 deg head tilt) thresholds. Thus, the authors concluded that nGVS appears to primarily impact otolith-mediated perception while having little impact upon canal-mediated perceptual thresholds. Notably, this study only investigated improvements with one subject-specific optimal nGVS application (as determined using a balance test, which non-specifically requires canal-otolith integration).

Here, we further pursue this question, by assessing vestibular perceptual thresholds across a range of nGVS amplitudes, enabling us to identify the presence (or lack thereof) of the characteristic SR response pattern. (See the Discussion for further details, but here we briefly summarize the difference in identifying improvements with one subject-specific optimal nGVS amplitude vs. testing with various nGVS amplitudes. A significant improvement with a single amplitude of nGVS (as compared to a no GVS/sham condition) is unable to demonstrate the *mechanism of SR*, since such an improvement could be due to confounding impacts of the presence of GVS, such as increased alertness, tactile sensations at the electrodes, reweighting of vestibular vs. non-vestibular cues, etc., either consciously or subconsciously, even if subjects do not notice the nGVS being applied. By testing multiple nGVS amplitudes, it is possible to identify (or fail to identify) the characteristic SR response pattern, which provides strong evidence for the mechanism of SR leading to improvements with an optimal amplitude of nGVS.) We studied the effect of nGVS on vestibular perception specifically targeting two motion “types” that primarily stimulated: (1) the otoliths (Y-translation or inter-aural using 1 Hz stimuli), and (2) the semicircular canals (Earth-vertical roll rotation at 0.2 Hz), and compared to perception that targeted both canal and otolith vestibular sensors (upright, Earth-horizontal roll tilt at 0.2 Hz, Galvan-Garza et al. 2018).

## Materials and methods

### Subjects

**Fig. 1:**
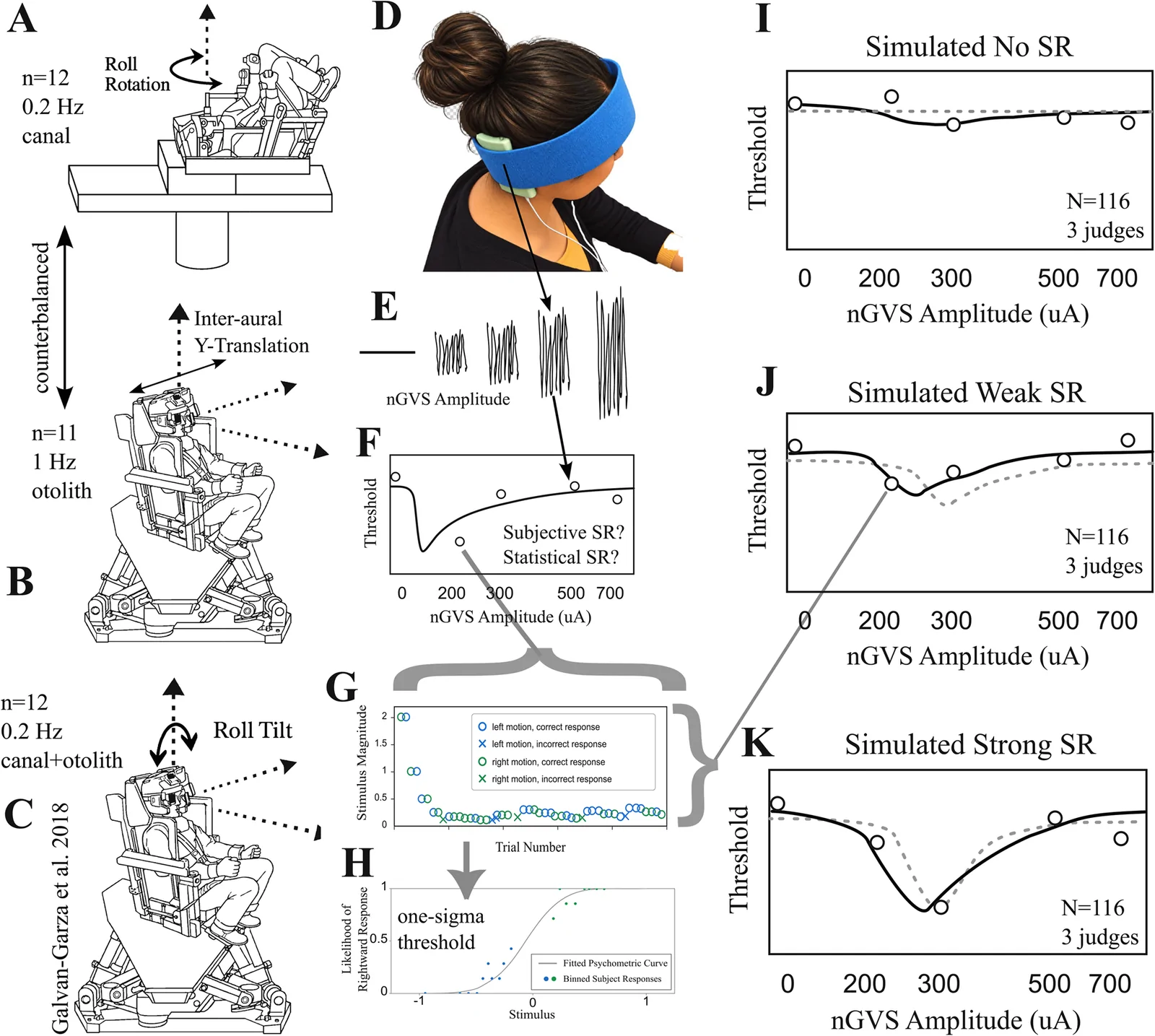
Summary of Methods. Three motion types were assessed: roll rotation at 0.2 Hz stimulating the semicircular canals (**A**), inter-aural Y-translation at 1 Hz stimulating the otoliths (**B**), and from Galvan-Garza et al. 2018 roll tilt at 0.2 Hz stimulating the canals and otoliths (**C**). Electrodes for noisy galvanic vestibular stimulation (nGVS) were applied throughout a session (**D**). Various nGVS current amplitudes were applied (**E**), during each of which thresholds were measured (**F**), a SR curve fitted (black), and judged to be subjective SR or further, statistical SR. Each threshold was measured using an adaptive staircase adjusting stimulus magnitude and forced-choice direction recognition task (**G**, with fewer than the full 150 trials shown for ease of visualization). Responses were fit with a psychometric curve to determine the “one-sigma” threshold (**H**). To compare to the empirical SR assessments, we performed simulations of no SR (**I**), weak SR (**J**), and strong SR (**K**), where the underlying SR response (gray) was sampled using the same adaptive staircase and threshold fitting. The measured thresholds (black circles) were fit with an SR curve (black) which is typically similar but not identical to the underlying SR curve due to measurement variability. The simulations (without the underlying SR response shown) were also judged, with the proportion of SR exhibitors compared to that found empirically for roll rotation, inter-aural translation, and roll tilt motion types

This study assessed 12 participants (6 female) who were healthy, had no known vestibular disorders with average (± one standard deviation) age of 26.6 ± 3.3 years. Seven of these subjects also participated in our previous roll tilt study (Galvan-Garza et al. 2018), based upon availability. Approximately a year had passed between the two experiments, such that aging effects upon vestibular thresholds (Bermudez-Rey et al. 2016) were likely not a factor. The inter-aural translation dataset consisted of 11 out of the 12 subjects because one subject, after completing the supine roll rotation tests, became medically ineligible to continue participating in experiments for unrelated reasons. Subjects signed informed consent forms and filled out a health screen questionnaire through the REDCap tool (Harris et al. 2009) hosted at the Massachusetts Eye and Ear Infirmary (MEEI). All testing took place in the Jenks Vestibular Physiology Laboratory (JVPL) at the MEEI. Figure 1 summarizes the motion conditions (A-C), experimental methods (D-H), and simulations used for comparison (I-K).

### Measuring vestibular perceptual thresholds with nGVS

Vestibular perceptual thresholds (Fitze et al. 2024) were measured and analyzed using the same methods previously developed (Merfeld 2011; Lim and Merfeld 2012; Chaudhuri and Merfeld 2013) and described with the application of nGVS (also referred to as SVS) in Galvan-Garza et al. (2018), differing only in the types of motion (Fig. 1A, B,C). At the beginning of each test session, electrodes were applied (Fig. 1D, Nuprep gel to prepare skin and cleaned with alcohol wipes, 5.1 × 10.2 cm UltraSim Electrodes placed on mastoids with generous layer of Signagel electrode gel) and impedance compliance (less than 1 kΩ) was assured. Then 5 motion threshold tests were conducted, each with 150 trials and a single nGVS amplitude (± 0, ± 200, ±300, ± 500, or ± 700 µA, peak to peak, Fig. 1E, F) applied continuously throughout the test session, in random order.

During test sessions, participants were secured with a five-point harness and adjustable head restraint. To minimize non-vestibular cues, testing was done in the dark and subjects wore headphones that played auditory white noise during motions. On each of the 150 trials, the motion direction (e.g., left vs. right) was randomized, following a single-cycle sinusoid of acceleration profile (Grabherr et al. 2008; Diaz-Artiles et al. 2017). After each motion, subjects performed a direction recognition forced-choice task, in which they indicated their perceived direction of motion by tapping a left or right button. Following a pause of at least three seconds, the next trial began. The magnitude of the motion (peak velocity) varied using an adaptive staircase (Fig. 1G).

At each nGVS amplitude, the vestibular perceptual threshold was determined by fitting a bias-reduced generalized linear model (MATLAB function brglmfit.m, Chaudhuri and Merfeld 2013), applying a cumulative Gaussian psychometric curve in which the standard deviation is the “one-sigma” threshold (Fig. 1H). This threshold definition corresponds to the magnitude of motion stimulus which can be distinguished (left vs. right) 84.1% correctly. The standard error of each “one-sigma” threshold was quantified using jackknife, leaving one trial out, refitting the threshold, to infer the uncertainty (Quenouille 1956; Tukey 1958), as previously done (Clark et al. 2024). Translation thresholds are reported in cm/s as the peak linear velocity of the stimulus, analogous to how rotation thresholds are reported in deg/s (Allred and Clark 2023). The translation thresholds expressed in acceleration amplitude (* A*) and net displacement (* p*) are proportional to peak velocity ($\:{v}_{max}$). For the 1 Hz translation motions, thresholds can be expressed in cm/s^2^ and cm, respectively: $\:A=\pi\:{v}_{max}$, $\:p={v}_{max}/\left(2\right)$, (Grabherr et al. 2008; Diaz-Artiles et al. 2017). In our previous study (Galvan-Garza et al. 2018), vestibular perceptual thresholds were assessed for upright, roll tilt about an Earth-horizontal axis with 0.2 Hz trajectories (5 s duration), a task that requires the integration of semicircular canal and otolith cues (Fig. 1C). In this study, subjects were tested in two sessions. In one session, subjects were tested on a Moog hexapod motion platform to create inter-aural Y-translation motions at 1 Hz (Fig. 1B), stimulating the otoliths without any stimulation to the canals. In the other session, subjects were tested on our Eccentric Rotator Device in the supine position in head-centered roll rotation about the Earth-vertical axis with 0.2 Hz motions (Fig. 1A), stimulating the canals without any change in stimulation to the otolith organs. The order of motion types was counterbalanced, such that half of the subjects were tested in inter-aural translation during the first test session.

None of our subjects reported sensations of motion, discomfort, or awareness of the electrical stimulation during the application of nGVS at any amplitude. While vestibular perceptual thresholds were assessed in the dark, subjects were given short breaks with light and were removed from the motion devices intermittently to maintain alertness. Subjects were repeatedly encouraged to report if they were too sleepy or “missed” a trial (occurred very infrequently), which then allowed us to repeat the trial with the direction re-randomized. Total testing time, including setup and breaks, was approximately 3.5 h for the supine roll rotation session and 3 h for the upright inter-aural translation session.

### Stochastic resonance determination analysis methods

Like in Galvan-Garza et al. (2018) and by others (Assländer et al. 2021), vestibular perceptual thresholds measured with each of five amplitudes of nGVS were fit with the theoretical SR equation (Benzi et al. 1981). We then used two criteria for SR, “Subjective SR” and “Statistical SR” as previously described (Galvan-Garza et al. 2018). In this study, one author provided subjective SR judgements on the experimental data (Fig. 1F), identifying which datasets subjectively matched the characteristic bell-shaped curve typically associated with SR. All individual datasets are shown to keep the author’s subjective judging transparent to the reader.

In addition to “subjective SR”, we also employed a more rigorous criterion which we call “statistical SR”. Statistical SR required both the presence of subjective SR, as well as a statistically significant decrease in the measured vestibular perceptual threshold from the sham (no nGVS applied) to the lowest vestibular perceptual threshold measured with any of the amplitudes of nGVS. We note that selecting the lowest measured threshold from four nGVS amplitudes elevates the false positive rate. This is mitigated by the additional requirement of subjective SR, but does not guarantee a specific false positive rate. This statistical evaluation used a one-tailed t-test (assuming only lower thresholds with nGVS vs. baseline), based upon the standard errors associated with threshold estimate. Nominally a cutoff of *p* < 0.05 was applied to match Galvan-Garza et al. (2018), however we had one case (S12 for roll rotation) which was marginal (*p* = 0.075). Rather than discard this case, we relax our criterion to *p* < 0.08 and report outcomes with both cutoffs.

Additionally, we used Monte Carlo simulations of thresholds with varying degrees of underlying SR to better understand the extent to which SR may appear to be exhibited despite not being present (and vice versa), due to the measurement variability associated with estimating thresholds with a finite number of trials. The same set of simulations and associated blinded judgements of SR from our previous study were used as representations of no (Fig. 1I), weak (Fig. 1J, defined by having an underlying improvement of 14.9% at the optimal nGVS amplitude compared to baseline), and strong SR (Fig. 1K, mprovement of 46%). In both SR cases the optimal nGVS amplitude was simulated as 300 µA using the theoretical SR curve (Benzi et al. 1981) and in all cases the baseline underlying threshold was 0.34 – unitless in simulations. Thresholds were simulated with the same number of trials and adaptive staircase as in the experiment, as well as fit with the same psychometric curve to estimate the thresholds (which would be similar, but not the same as the underlying threshold at a given nGVS amplitude due to measurement variability). The simulated threshold responses were visualized and blindly judged as exhibiting SR or not. Since the simulated dataset were known to have underlying strong, weak, or no SR, this provided a means of controlling for false positive identification of SR when in fact no underlying SR had been simulated.

The proportion of SR exhibitors within each experimental subject group was compared to the set of simulations to assess whether SR exhibition was present more or less than what was expected to occur by chance. To assess whether SR was exhibited on only otolith-mediated or only canal-mediated vestibular perceptual thresholds, we compared the proportion of subjects that had significantly lower vestibular perceptual thresholds with nGVS than without nGVS across the three motion types tested: upright inter-aural Y-translation (otolith-mediated), supine roll rotation (canal-mediated), and upright roll tilt (from the previous Galvan-Garza et al. 2018 study, stimulated the canals and otoliths).

## Results

### Supine roll rotation thresholds stimulating the semicircular canals

**Fig. 2:**
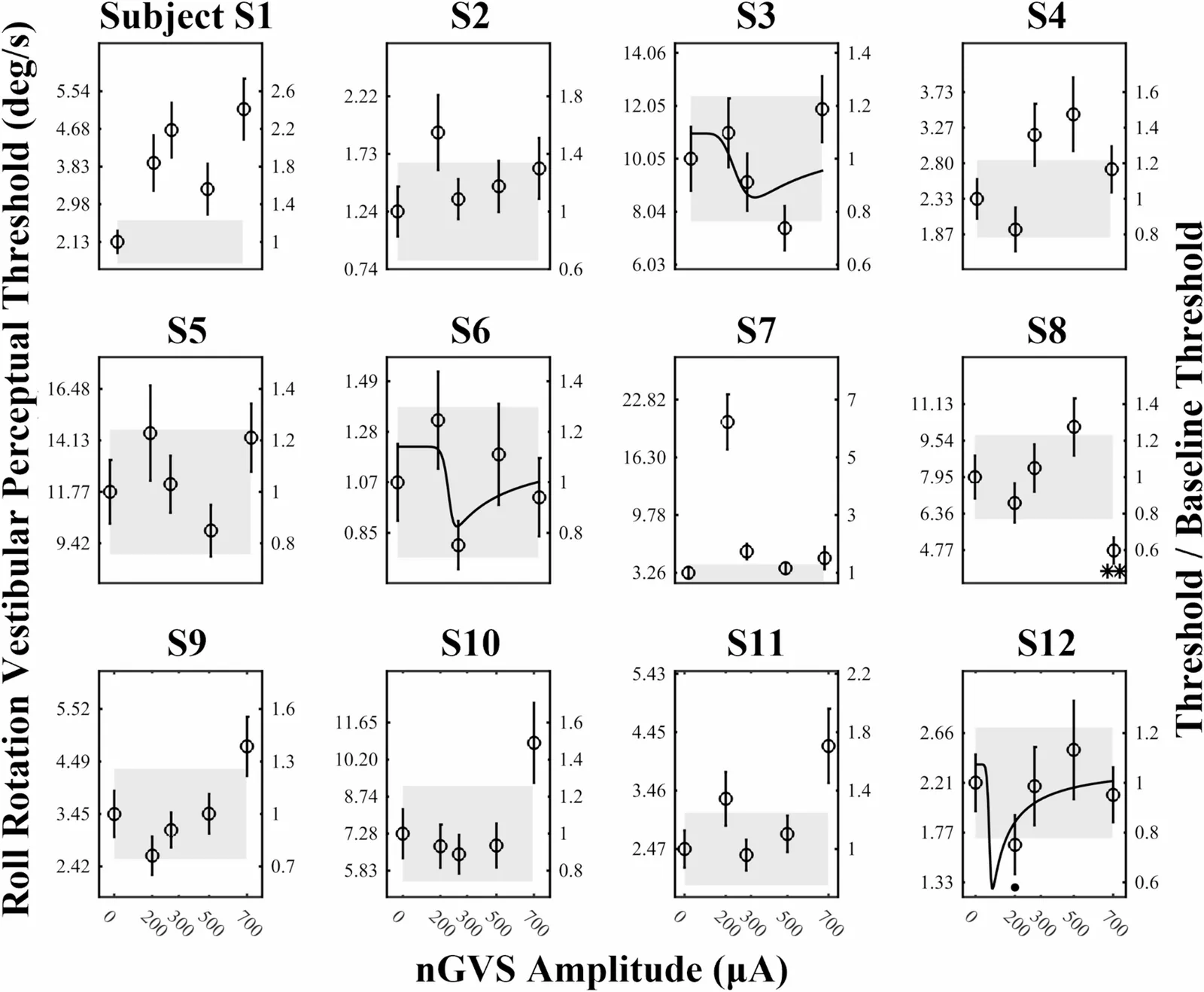
Supine roll rotation vestibular perceptual thresholds vs. nGVS amplitude. Left axis indicates the roll rotation threshold in deg/s. Right axis indicates the threshold normalized by the baseline threshold (with 0 µA or no nGVS being applied). Error bars indicate standard error of the threshold estimate. Shaded areas indicate the 95% confidence interval of the baseline threshold (extended across the plot in a band to allow for comparison to the thresholds at each nGVS amplitude). Overlaid line is the fit SR curve for each individual subject, when a successful fit was possible. p-values refer to a statistically significantly lower roll rotation threshold compared to the baseline (0 µA) threshold. ** *p* < 0.005, * *p* < 0.05, • *p* < 0.08

As detailed above, we employed two criteria for SR exhibition: (1) subjective SR, by visualizing the pattern of thresholds and its consistency with the theoretical SR curve response, and (2) statistical SR, which in addition required a significantly lower threshold with the optimal nGVS amplitude, as compared to the baseline threshold (i.e., that with no GVS applied, or sham). In terms of subjective SR, only 3 out of 12 subjects met this criterion (25% of subjects: S3, S6, and S12) for vestibular perceptual thresholds in the supine roll rotation motion type, primarily stimulating the canals (Fig. 2). For the three subjective SR exhibitors, the difference between minimum and baseline threshold ranged from − 0.55 deg/s (S12) to -2.65 deg/s (S3). In percent change from baseline, the range was − 24.8% (S6) to -26.3% (S3).

**Fig. 3:**
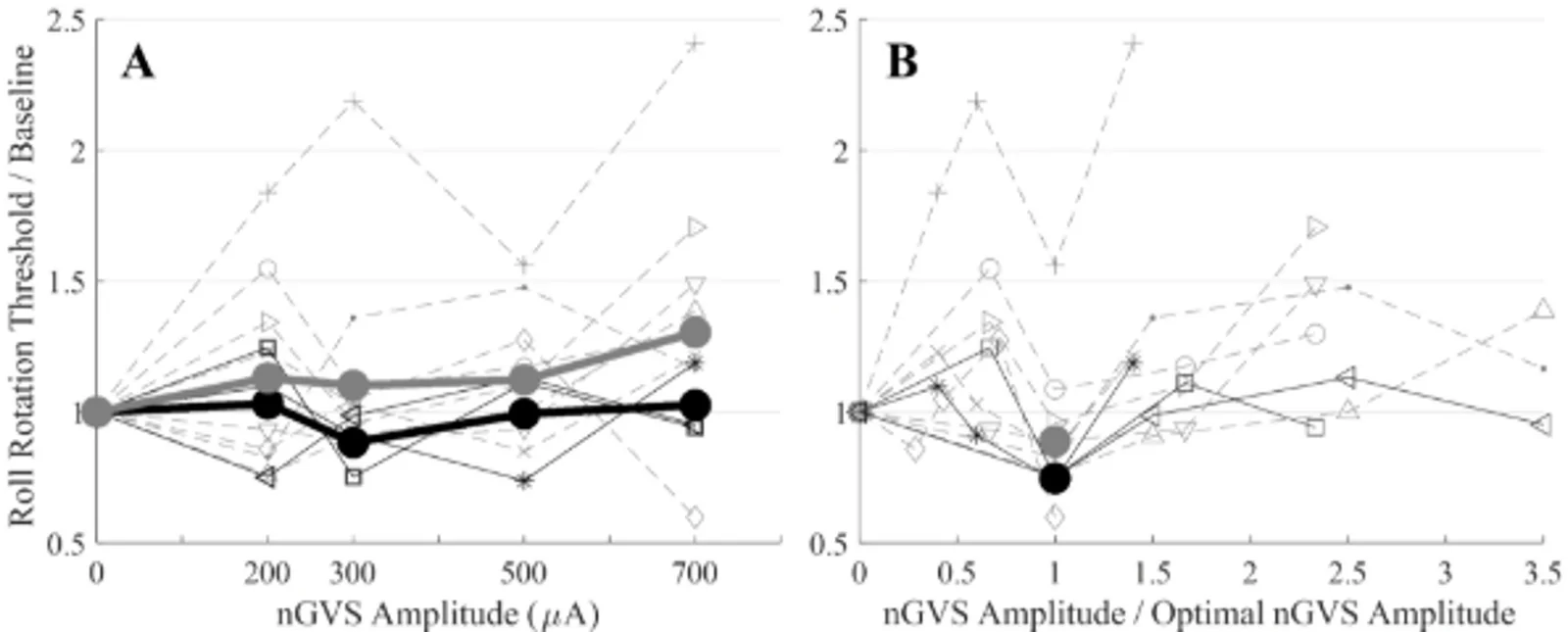
Group supine roll rotation vestibular perceptual thresholds. **A**: Shown in thin lines and open symbols are each individual subject’s thresholds normalized by their baseline threshold vs. nGVS amplitude applied. Subjects which exhibited subjective SR are shown with solid black lines, while others are in dotted gray lines. The thick lines and filled circles are the average across subjects, with the thick gray line being the average of all subjects, and the thick black line being the average of only the subjects exhibiting subjective SR. **B**: The same thresholds normalized by baseline vs. nGVS amplitude, now normalized by each subject’s nGVS amplitude which yielded the minimum threshold (excluding the baseline with no nGVS). This visualization scales the x-axis for a value of 1 aligning to each subject’s individualized optimal amplitude of nGVS. S7 is not shown because of an extreme outlier in their dataset (see Fig. 2)

For roll rotation thresholds, only one subject (1/12, 8%, S12) marginally exhibited statistical SR (*p* = 0.075 for significance, while demonstrating subjective SR). (While by our earlier cutoff of *p* < 0.05 even this one case would not have been considered statistical SR, we note it here. Had this case been discarded as not exhibiting statistical SR, the conclusion that roll rotation thresholds rarely exhibit SR becomes even more definitive.) A general lack of SR exhibition was also evident in the group data for roll rotation thresholds, shown in Fig. 3.

Across 11 subjects (S7 excluded because of extreme outlier), and when the roll rotation thresholds were normalized by each individual’s baseline threshold, the lowest average roll rotation threshold actually occurred when 0 µA nGVS (i.e., sham) was applied (Fig. 3A). Alternatively, when the nGVS amplitude was normalized (on the x-axis) by each subject’s nGVS amplitude which yielded the minimum threshold (Fig. 3B), these normalized thresholds were 11.2% less than baseline. This decrease of 0.842 deg/s was statistically significant (paired t(10) = 2.220, *p* = 0.025). However, this highlights the importance of not just comparing the “optimal” threshold to each subject’s baseline, because with measurement variability, false positives may occur. Additionally, two of the subjects shown in Fig. 3 had their minimum threshold at the baseline (0 µA nGVS applied). Therefore, we conclude that SR was not evident in the primarily semicircular canal-mediated, supine roll rotation threshold data.

### Upright inter-aural translation thresholds stimulating the otolith organs

**Fig. 4:**
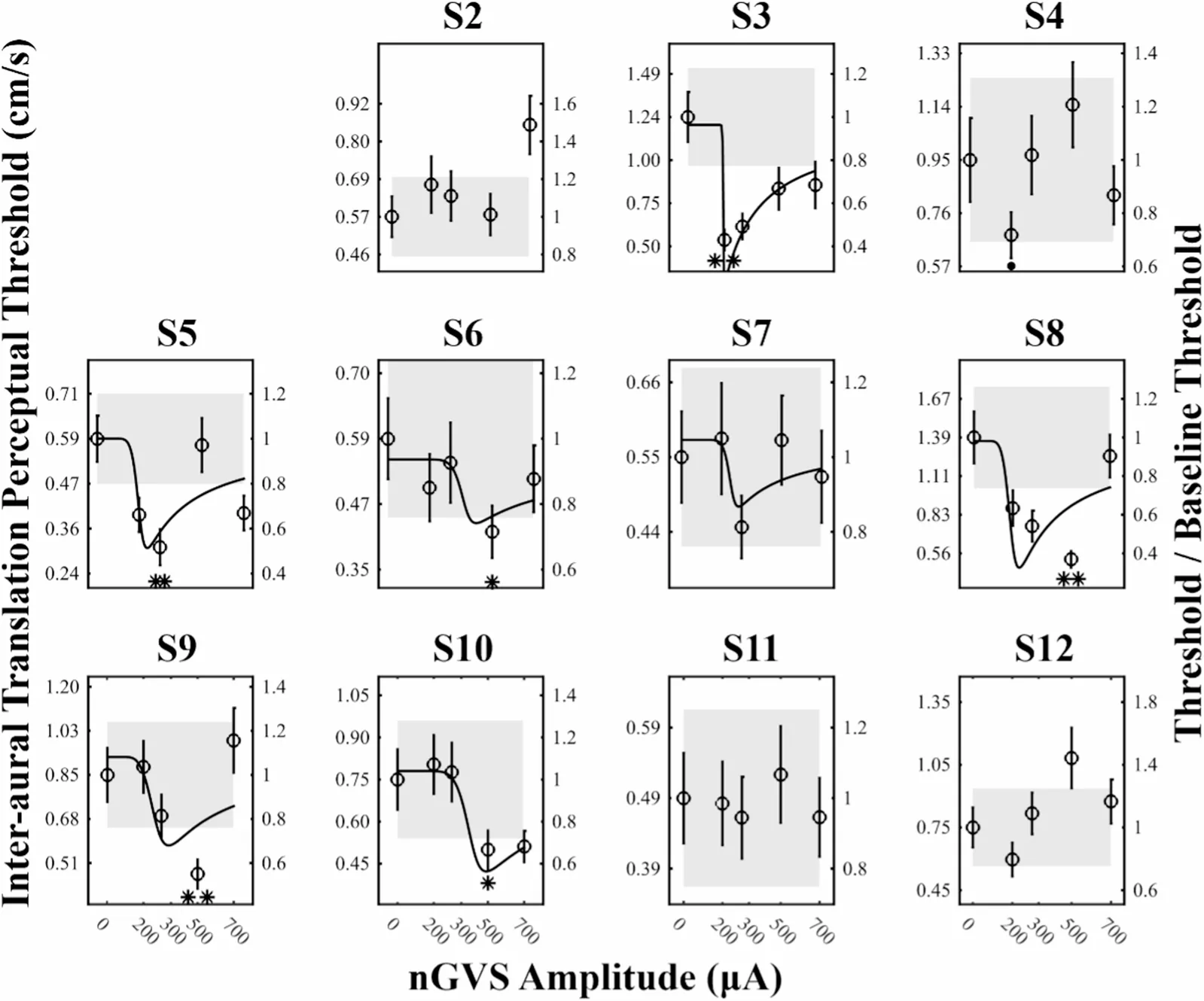
Upright inter-aural Y-translation thresholds (1 Hz) vs. nGVS amplitude. Figure specifics are identical to those in Fig. 2. p-values refer to a statistically significantly lower threshold compared to the baseline. ** *p* < 0.005, * *p* < 0.05, • *p* = 0.08

In contrast, for inter-aural translation thresholds, subjective SR was exhibited in 7 out of 11 (64%) subjects (S3, S5, S6, S7, S8, S9, S10, Fig. 4). Additionally, 6 out of the 11 subjects also had statistical SR, with a significantly lower threshold than baseline (S3, S5, S6, S8, S9, S10, indicated in Fig. 4). For the subjective SR exhibitor group, the change in threshold due to the “optimal” nGVS amplitude ranged from − 0.9 cm/s (S8) to -0.2 cm/s (S6) and in percent change from baseline, the range was − 63.1% (S8) to -18.7% (S7), with an average of -42%. For the subset reaching the statistical SR criteria, the smallest difference from baseline and percent change were − 0.17 cm/s (S6) and − 28.5% (S6), respectively, with an average percent change of -46%. Additionally, across all subjects, out of all thresholds measured with nGVS applied, only two (from S2 and S12) were above the 95% confidence interval of the baseline and both of these subjects were identified as non-responders.

**Fig. 5:**
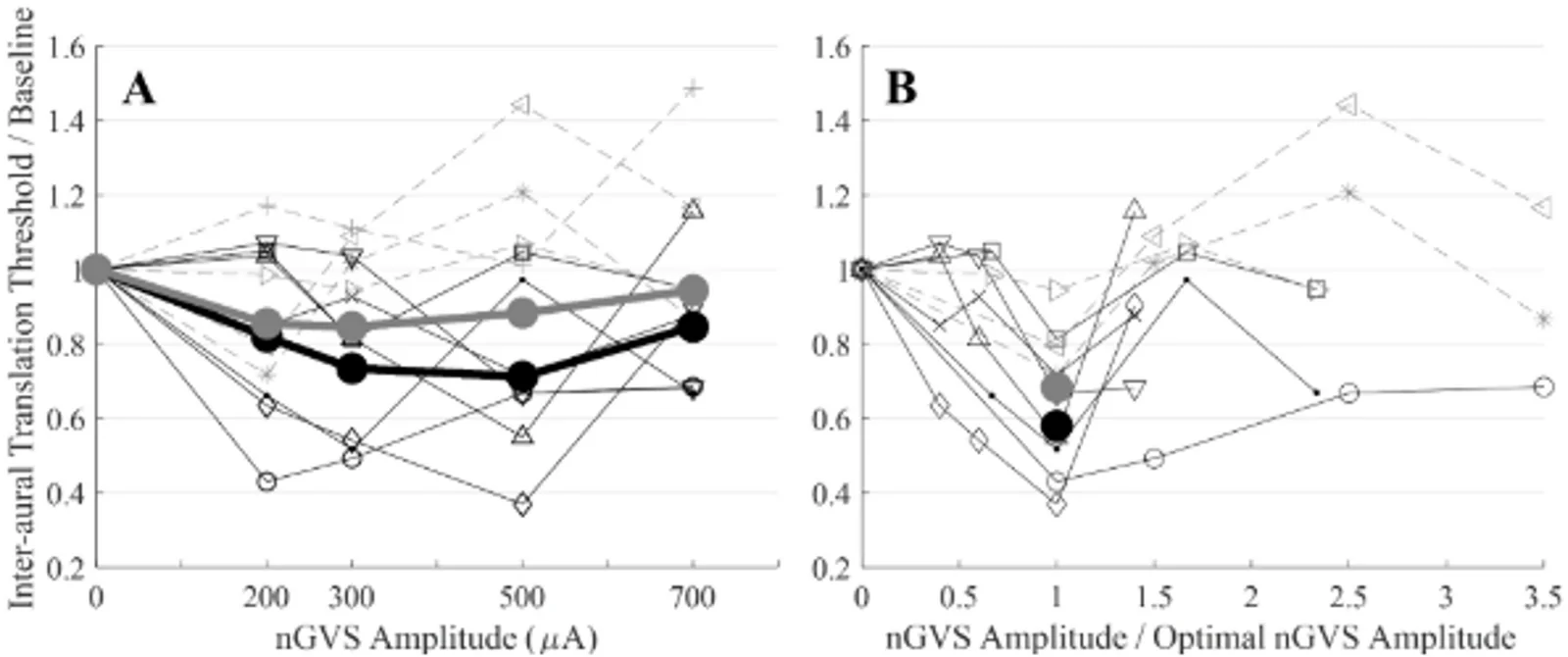
Group upright inter-aural y-translation vestibular perceptual thresholds. Figure specifics are identical to those in Fig. 3

When the inter-aural thresholds from all 11 subjects were averaged together, the lowest average motion thresholds were achieved at 300 µA nGVS amplitude, with a normalized change from baseline of 15.4% (Fig. 5A), corresponding to a decreased threshold of 0.2 cm/s (paired t(10) = 1.894, *p* = 0.044). The normalized change was 31.7% between the group average baseline and subject-specific minimum threshold with nGVS (Fig. 5B), corresponding to a statistically significant decrease of 0.318 cm/s (paired t(10) = 3.24, *p* = 0.005, though as noted above for roll rotation thresholds this subject-specific selection of minimum threshold across nGVS amplitudes can produce false positives).

With the four presumed non-SR exhibitors removed from the dataset, the group optimal nGVS amplitude was 500 µA with a normalized change of 26.6% from baseline (Fig. 5A). This was a statistically significant decrease of 0.34 cm/s (paired t(6) = 2.633, *p* = 0.020). There was also a statistically significant decrease of 0.30 cm/s between the baseline and 300 µA nGVS group means (paired t(6) = 2.305, *p* = 0.031). Finally, the inter-aural threshold at the subject-specific optimal nGVS amplitude had a normalized improvement from baseline of -42% (Fig. 5B), corresponding to statistically significant decrease of -0.44 cm/s (paired t(6) = 3.282, *p* = 0.009).

### Comparison of experimental data to simulated data

**Fig. 6:**
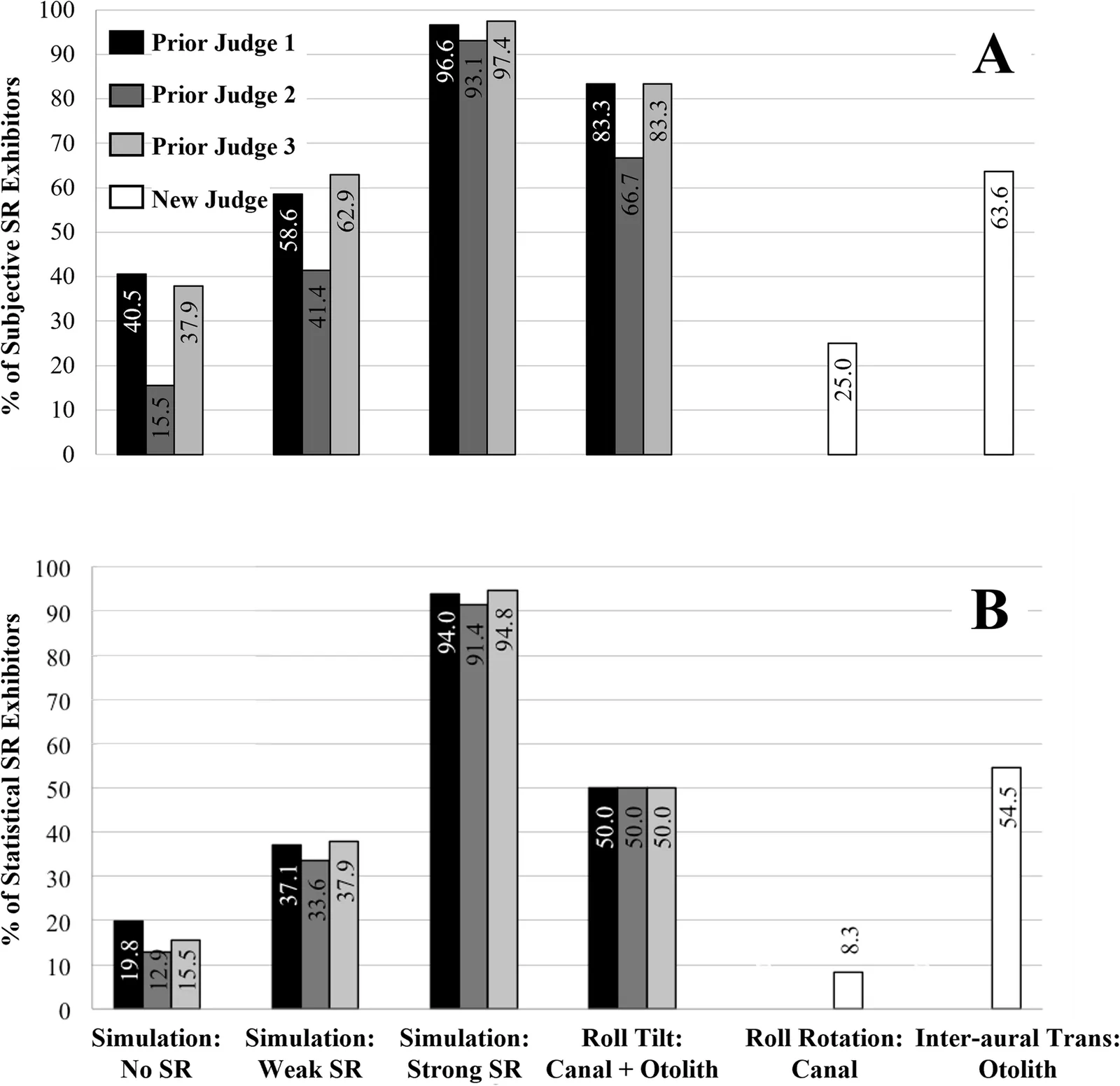
Percentage of subjects judged to be SR exhibitors based upon the criteria of subjective SR (panel **A**) and the more rigorous statistical SR (panel **B**). Adopted from Galvan-Garza et al. 2018, prior simulated datasets were blindly judged by three judges, in which there was no underlying SR, weak underlying SR (underlying improvement of 14.9%), and strong underlying SR (improvement of 46%). In each case, *N* = 116 simulations were used. These serve as references to compare empirical outcomes to, in terms of the proportion of subjects exhibiting SR. The outcome of the three prior judges’ assessments for roll tilt thresholds are reproduced from Galvan-Garza et al. 2018. For the current data sets, a single new judge assessed SR exhibition for roll rotation and inter-aural translation, which are shown with white bars on the right

The proportion of subjective SR exhibitors (3/12 = 25%) in supine roll rotation was not statistically significantly different than that of the simulation group with No underlying SR (z=-0.45, *p* = 0.326) and was statistically lower than those of the Weak (z=-1.93, *p* = 0.027) and Strong underlying SR (z=-7.47, *p* < 0.001) simulation groups (Fig. 6). Similarly, the proportion of subjects with statistical SR (1/12 = 8.3%) was not statistically significantly different than that of the simulation group with No underlying SR (z=-0.71, *p* = 0.239) and was statistically lower than those of the Weak (z=-1.95, *p* = 0.025) and Strong underlying SR (z=-8.04, *p* < 0.001) simulation groups. The same conclusions are obtained if the single subject is deemed to not exhibit statistical SR by the strict *p* < 0.05 criterion. This suggests that the exhibition of SR (subjectively and statistically) in this supine roll rotation data set did not occur more than what would be expected to occur by chance.

The proportion of subjective SR exhibitors in inter-aural translation (7/11 = 64%) was statistically significantly higher than that of the corresponding simulation group with No underlying SR (z = 2.16, *p* = 0.015), not different from that of the Weak underlying SR simulations (z = 0.59, *p* = 0.278), but lower than that of the Strong underlying SR simulations (z=-3.96, *p* < 0.001). Similarly, the proportion of statistical SR exhibitors (6/11 = 55%) was also significantly higher than No underlying SR (z = 3.08, *p* = 0.001), not different than Weak SR (z = 1.20, *p* = 0.115), but lower than Strong SR (z=-4.20, *p* < 0.001).

### Within subject comparisons of SR exhibition across motion types

It was not an original goal of this research to compare SR exhibition within individuals, across upright roll tilt (previous study, Galvan-Garza et al. 2018), supine roll rotation and inter-aural translation (this study) motion types. However, there were seven subjects that were tested in both studies, enabling a comparison for this subset of subjects (Table 1).

When referencing the subject numbers from this study in Table 1, there was one subject who was judged to have subjective SR in all three motion directions (S6 in this study, S3 in the prior study). Five out of the six subjects that were tested in both upright roll tilt and inter-aural translation exhibited SR in both tests (only S12 exhibited SR for upright roll tilt, but not inter-aural translation). On the other hand, of the seven subjects that exhibited SR for upright roll tilt, only two of them exhibited subjective SR for supine roll rotation (S6 in this study/S3 in the prior study and S12). Similarly, of the four subjects that exhibited SR for inter-aural translation, none of them did for roll rotation (note that S12 exhibited SR for roll rotation, but not for translation). Thus from this limited dataset, across motion types it does not appear that some subjects were “SR exhibitors” across motion types and others were not, but instead that most subjects exhibited SR for upright roll tilt and inter-aural translation, and not for supine roll rotation.

Next, we considered if the optimal nGVS amplitude was consistent across motion types, when a subject exhibited SR. There was little evidence of such consistency within each subject; while S6’s (S3 in the prior study) optimal nGVS amplitude was 500 𝝁A for roll tilt and translation thresholds, it was 300 𝝁A for roll rotation, and the other five subjects had different optimal nGVS amplitudes across the two motion types in which they exhibited SR.

Finally, we tested if a subject improved more so (or less so) with nGVS for roll tilt, was that related to the amount of improvement in their translation threshold. We found that, no, the percentage difference between baseline and optimal threshold in upright roll tilt and inter-aural translation tests was not positively correlated across subjects.

## Discussion

### Summary of primary findings

In this study we investigated whether SR was exhibited in vestibular perceptual thresholds during a motion type that predominantly stimulated the semicircular canals or otolith organs to gain a better fundamental understanding of how vestibular SR occurs. Noise was added to the vestibular system via the application of subsensory bilateral bipolar galvanic vestibular stimulation. For supine roll rotation thresholds (primarily stimulating the canals), we found that the proportion of “subjective” SR exhibitors (3/12) was no different than could occur by chance (i.e., if there was No underlying SR) and was significantly lower than what would be expected from the Weak and Strong underlying SR simulations. There was only one subject (in fact, zero by the strict definition of *p* < 0.05) who was judged to exhibit “statistical” SR (subjective SR with a statistically significantly lower threshold than baseline) which again could have statistically occurred by chance.

However, for inter-aural translation thresholds (primarily stimulating the otolith organs), subjective vestibular SR was exhibited at a proportion (7/11 subjects) significantly higher than what could occur by chance, and consistent with simulated Weak SR. The proportion of subjects that also had a significantly lower than baseline threshold (6/11) was statistically similar to the proportion of Weak SR simulated subjects. Although SR was exhibited in the primarily-otolith translation threshold task, the rate of exhibition was slightly less than that for upright roll tilt thresholds (9/12 subjects in our previous study, Table 2), when both the canals and otoliths were stimulated. We conclude that the exhibition of stochastic resonance in vestibular motion perception thresholds may be unique to motion types which involve stimulation of the otolith organs.

**Table 2:** Summary of experimental and simulated vestibular perceptual thresholds with nGVS results in ascending order of subjective SR exhibition

Group	Frequency (Hz)	Predominantly stimulates	Subjective SR Exhibition	Statistical SR Exhibition
Supine Roll Rotation	0.2	canals	3/12 (25%)	1/12 (8%), 0/12 (0%) by the strict *p* < 0.05 criterion
*Simulations – No SR*	-	-	31%	16%
*Simulations – Weak SR*	-	-	54%	36%
Inter-aural translation	1	otolith organs	7/11 (64%)	6/11 (55%)
Upright Roll Tilt	0.2	canals & otolith organs	9/12 (75%)	7/12 (58.3%), 6/12 (50%) by the strict *p* < 0.05 criterion
*Simulations – Strong SR*	-	-	96%	93%

We note one nuance in Table 2’s statistical SR outcomes. In Galvan-Garza et al. (2018) we strictly applied a statistical criterion of *p* < 0.05 for each subject’s threshold to exhibit statistical SR. In the current dataset, for roll rotation there was one case which neared this criterion (*p* = 0.075). Rather than discard this case and conclude no participants demonstrated statistical SR, we note it as being less than *p* < 0.08 (Fig. 2). If we retrospectively apply that same relaxed criterion to the upright roll tilt thresholds (Galvan-Garza et al. 2018) for a fair comparison, 7/12 (rather than 6/12) participants exhibited statistical SR. Table 2 notes both outcomes and this does not impact our conclusions.

### Comparison to previous studies

As noted earlier, Keywan et al. 2019 investigated the role of the semicircular canals vs. otolith organs in nGVS improving vestibular perceptual thresholds, which our study now advances in the following ways. Most importantly, Keywan et al. 2019 tested a single amplitude of nGVS (specific to each subject, based upon that which most reduced each subject’s postural sway in a 30 s test standing on foam with eyes closed) vs. a sham condition (no nGVS applied). They found significant improvements in otolith-mediated inter-aural translation thresholds, but no difference in canal-mediated rotational thresholds, consistent with our findings. However, our study investigated a suite of nGVS amplitudes (0, 200, 300, 500, and 700 𝝁A), enabling identification of the presence of SR exhibition (or the lack thereof) via the characteristic bell-shaped curve. As noted in the Introduction, improvements with a single amplitude of nGVS do not, in isolation, demonstrate the presence of SR, since this effect could be the result of other non-SR related mechanisms like increased alertness, changes to vestibular vs. non-vestibular weighting, and tactile sensations at the electrode site, even if subconsciously. In contrast, observing a characteristic SR “dip” as a function of nGVS amplitude strongly suggests the mechanism of SR. Thus, the current study is the first to show SR exhibition in vestibular perceptual thresholds is specific to otolith-mediated motion types and not those that are canal-mediated.

In addition, there were notable differences between the two studies. In terms of motion types, for their canal-mediated threshold test, Keywan et al. had the subject seated upright, but with the head pitched forward by 71 degrees to place the anterior and posterior canals (which nominally sense roll tilt with head upright) roughly oriented to sense the provided Earth-vertical yaw rotation (vs. our supine roll rotation with no head tilt). Both are canal-mediated motion types, but Keywan et al. adds head on neck tilt, while our roll rotation placed the subject supine, altering the whole-body, upright seated position used for inter-aural translation and roll tilt thresholds. Keywan et al.’s yaw rotation was performed at 1 Hz (motion duration of 1 s) matching the inter-aural translation condition, while we used 0.2 Hz roll rotation (duration of 5 s) to match the roll tilt thresholding.

In terms of nGVS waveform, Keywan used a 0–2 Hz bandlimited stimulation, while we used broadband 0–30 Hz. While both 0–2 Hz (Keywan et al. 2018, 2019) and 0–30 Hz (Galvan-Garza et al. 2018; Mulavara et al. 2011a, b; Temple et al. 2018; Goel et al. 2015) have been used previously, it is possible that one nGVS waveform may be more effective at inducing SR, or may be so for certain motion types or frequencies (e.g., 1 Hz rotation vs. 0.2 Hz rotation or tilt). To our knowledge, no study has investigated the impact of more than one nGVS waveform on vestibular perceptual thresholds, though postural sway was found to be improved roughly similarly with both 1–2 Hz and 0–30 Hz nGVS waveforms (Mulavara et al. 2011a, b) and a review also found postural benefits using various nGVS waveforms (McLaren et al. 2023).

Lastly, in addition to Keywan et al. 2019; we aim to put the current study in the context of the findings from Keywan et al. 2018. This earlier paper investigated roll tilt thresholds across three motion frequencies, identifying significant improvements with a single subject-specific nGVS amplitude (determined using the same postural sway test) for 0.5 and 1 Hz, but not 0.2 Hz roll tilts. This conflicts with the conclusion that nGVS improves otolith-mediated vestibular perceptual thresholds, but not those motion types which are canal-mediated. While roll tilt stimulates both the canals and otolith organs, roll tilt thresholds are thought to be determined by otolith sensitivity at lower frequencies, since the angular velocity of the tilt motion is small and at lower frequencies is poorly transduced by the canals which have high pass filter dynamics. However, higher frequency (above about 0.3–0.5 Hz) roll tilt thresholds are mostly determined by canal sensitivity due to larger peak and higher frequency angular velocity stimulation (Lim et al. 2017). Thus, if nGVS benefits are otolith-mediated, we would have expected to see improvements for 0.2 Hz roll tilt, but less so or no improvements at 0.5–1 Hz roll tilt (which are more canal-mediated); however, Keywan et al. 2018 reports the exact opposite. Future testing is needed to properly address this inconsistency, so here we can briefly speculate. First, it may be related to how the subject-specific optimal nGVS amplitude was selected in Keywan et al. 2018 and 2019; through assessing postural sway. The nGVS amplitude which reduced sway the most for an individual may be effective for improving higher frequency vestibular perceptual thresholds (roll tilt 0.5 and 1 Hz and inter-aural translation 1 Hz), but another amplitude may be necessary for the lower frequency (roll tilt at 0.2 Hz). Future work should explore nGVS improvements in vestibular perceptual thresholds alongside postural sway, using a broad set of nGVS amplitudes to identify the “optimal” for each task.

Next, the inconsistency may also be due to an interaction of the nGVS waveform, with Keywan et al. 2018 and 2019 both using 0–2 Hz bandlimited nGVS, while Galvan-Garza et al. 2018 and the current study applying broadband 0–30 Hz nGVS. Again, future work should directly compare the efficacy of different nGVS waveforms (0–2 Hz vs. 0–30 Hz, etc.) towards producing SR.

Finally, it should be acknowledged that it is possible the reported improvements in Keywan et al. 2018 were false positives, with *p* = 0.045 for 0.5 Hz and *p* = 0.013 for 1 Hz, without mention of a correction for the multiple comparisons testing 3 frequencies. Similarly, it is possible that the lack of improvement for roll tilt at 0.2 Hz was a statistical “miss”. After all, Galvan-Garza et al. 2018 reported 9 of 12 subjects exhibited SR in roll tilt thresholds at 0.2 Hz, suggesting an improvement would be expected.

### Summary of secondary findings

With only the subset of the seven subjective inter-aural translation SR exhibitors, there was a significant correlation between baseline motion threshold and the improvement in thresholds with optimal nGVS (baseline threshold – minimum nGVS threshold) (*R* = 0.958, *p* < 0.001). Similarly, there was a statistically significant correlation between baseline motion threshold and the percent change in threshold between baseline and minimum nGVS thresholds (*R*= -0.795, *p* = 0.003). Like in our previous study and in Keywan et al. 2018 and 2019; these results suggest that if a subject had relatively worse (i.e., higher) thresholds in the baseline without nGVS, they had more room for improvement with nGVS (both absolute and normalized by the baseline). We note, however, to interpret these relationships with caution since the baseline threshold measurement is being correlated with an outcome that is a function of that same baseline measurement (i.e., improvement = baseline threshold - minimum nGVS threshold).

While a secondary goal of this effort, we opportunistically considered (Table 1) the subset of subjects which were tested in more than one motion type (inter-aural translation at 1 Hz, supine roll rotation at 0.2 Hz, and in our previous study upright roll tilt at 0.2 Hz). We found little evidence for consistency within each subject across motion types, either in exhibiting SR or not, the optimal nGVS amplitude for improving thresholds, or the percentage of threshold improvement with nGVS. However, the sample sizes available for these analyses were very small (5–7 subjects) and the measurement variability was large in the metrics of optimal nGVS amplitude and percentage of threshold improvement (both are a function of multiple threshold measurements, each of which has moderate measurement variability, even when assessed with 150 trials). Even simply being judged as exhibiting SR or not has a substantial false positive rate and miss rate (see Fig. 6, where simulations with No underlying SR were incorrectly judged to exhibit SR ~ 20% of the time, while 7% of simulations with Strong underlying SR were incorrectly judged to not be exhibiting SR). Future work should aim to test a larger cohort of subjects to determine if some individuals tend to exhibit SR (or not) across vestibular perceptual threshold motion types, and whether the percentage improvement or optimal nGVS amplitude are consistent/related within an individual across motion types. The raw findings in Table 1 are provided, such that they can be incorporated into future analyses.

### Limitations of the current study

In addition to primarily stimulating the semicircular canals vs. the otolith organs (or both), we note other differences in the two motion types assessed, which could be confounding. Supine roll rotations were performed at 0.2 Hz to enable a direct comparison to the upright roll tilt motion thresholds measured in Galvan-Garza et al. 2018; which were also done at 0.2 Hz. Inter-aural translation thresholds were assessed at a commonly tested frequency of 1 Hz (Grabherr et al. 2008; Valko et al. 2012a, b; Lim and Merfeld 2012), instead of 0.2 Hz (i.e., each motion was 1 s in duration, rather than 5 s). As inter-aural thresholds increase at lower frequencies (Fitze et al. 2024) and thus require a larger motion range, it would not have been feasible to assess thresholds at 0.2 Hz given the capabilities of our motion platform. We further note that the otolith stimulation pattern during upright roll tilt is substantially different from that for inter-aural translation. Upright roll tilt stimulates both the utricle and saccule with the peak change in stimulation happening during the static tilt at the end of the motion, while inter-aural translation stimulates primarily the utricle with peak stimulation occurring during the middle of the dynamic translation motion. Thus, even if the frequency was matched between upright roll tilt and inter-aural translation, it would not replicate the otolith stimulation profile. Alternatively, “pseudo-static” roll tilt thresholds primarily stimulate the otoliths (both utricle and saccule) without stimulating the canals (Lim et al. 2017), by performing the tilts very slowly (< 1 degree/second of peak angular velocity). While these tests are tedious due to the long/slow tilts (e.g., require 1 h of testing at each nGVS amplitude), if SR is exhibited in pseudo-static roll tilt thresholds, it further supports our finding that vestibular SR occurs for motion types with predominantly otolith stimulation.

Our approaches for identifying SR through a combination of subjective judgements and statistical criterion, as compared to simulations, are an improvement upon prior approaches more susceptible to false positives or that only identify improvements, rather than SR responses. However, subjective judgements introduce another source of variation and nuance and even our strict statistical SR criterion does not guarantee a false positive rate of α = 0.05. Future work could leverage machine learning classification (Voros et al. 2022) to objectively identify SR.

It is also well known that vestibular function is highly frequency dependent. Our tests consisted of only two frequencies (0.2 Hz and 1 Hz), between the roll tilt and roll rotation vs. inter-aural translation thresholds. To our knowledge, no studies have investigated the impact of nGVS across frequencies for a given motion type that primarily stimulates either only the otolith organs (e.g., inter-aural translation thresholds at 1 Hz, 0.5 Hz, and 0.2 Hz) or only the canals. Keywan et al. 2018 investigated the effect of nGVS (single, subject-specific amplitude) on roll tilt thresholds, at 0.2, 0.5, and 1 Hz, however roll tilt stimulates both the canals and otoliths to varying degrees as a function of the frequency of tilt motion. Further testing over a range of motion frequencies, along with multiple motion types would provide the data needed to fully define the characteristics of vestibular perceptual SR due to bilateral bipolar nGVS application.

## Data Availability

The datasets generated during and/or analyzed during the current study are available from the corresponding author on reasonable request.
